# Investigative Approaches to Resilient Emotion Regulation Neurodevelopment in a South African Birth Cohort

**DOI:** 10.1016/j.bpsgos.2025.100457

**Published:** 2025-01-31

**Authors:** Tristan Yates, Siphumelele Sigwebela, Soraya Seedat, Michael Milham, Stefan du Plessis, Lior Abramson, Erica Niemiec, Carol Worthman, Mary Jane Rotheram-Borus, Giovanni Salum, Alexandre Franco, Arianna Zuanazzi, Fatima Ahmed, Kelly Gemmell, Joan Christodoulou, Nomandla Mhlaba, Noluncedo Mqhele, Nomfusi Ngalimane, Akhona Sambudla, Nim Tottenham, Mark Tomlinson

**Affiliations:** a Department of Psychology, Columbia University, New York, New York; b Institute for Life Course Health Research, Department of Global Health, Stellenbosch University, Cape Town, South Africa; c Department of Psychiatry, Faculty of Medicine and Health Sciences, Stellenbosch University, Cape Town, South Africa; d Center for the Developing Brain, Child Mind Institute, New York, New York; e School of Psychological Sciences, Tel Aviv University, Tel Aviv, Israel; f Department of Anthropology, Emory University, Atlanta, Georgia; g Semel Institute, Department of Psychiatry and Biobehavioral Sciences, University of California at Los Angeles, Los Angeles, California; h Department of Psychology, Palo Alto University, Palo Alto, California; i School of Nursing and Midwifery, Queens University, Belfast, Northern Ireland, United Kingdom

**Keywords:** Adolescent, Emotion regulation, Low- and middle-income country, MRI, Neurodevelopment, Resilience

## Abstract

Understanding the neurobiology of resilient emotion regulation following adversities is critical for addressing mental health problems globally. However, the functional neurobiology of resilience has rarely been studied in low- and middle-income countries, which comprise 90% of the world’s population and experience more consistent adversities. Here, we describe how we are investigating the neurodevelopment of resilient emotion regulation in adolescents (anticipated *N* = 525) from a South African birth cohort recruited from a low-income, high-adversity township. Across 2 longitudinal time points (13–14 and 15–16 years), magnetic resonance imaging, behavior, and self-report measures from adolescents and their caregivers are collected. These data are complemented by existing developmental histories (from the prenatal period to 8 years). The culturally adapted measures, protocols, and analytic plans for investigating resilient emotion regulation are presented. By characterizing neurodevelopmental correlates of adolescent resilience from an understudied low- and middle-income country, this research will provide deeper insights into mental health globally.

## Plain Language Summary

The functional neurobiology of resilient emotion regulation is understudied in adolescents from low- and middle-income countries. We describe our protocol for measuring the functional neurobiology that underlies resilient emotion regulation in a longitudinal cohort of South African adolescents from a high-adversity setting. These data will inform our understanding of neural mechanisms that underlie resilient emotion regulation and how adverse and protective factors affect those neurodevelopmental profiles in South African adolescents, with implications for global mental health.

Early-life adversity is a leading environmental risk factor for mental illnesses (1, 2, 3). This robust link has accelerated the study of factors associated with successful development and coping despite adversity exposure—in other words, resilience (4). Emotion regulation, or the ability to modulate the intensity and duration of positive and negative emotions, is a key psychological process related to positive developmental outcomes following adversity (5). Although in an ideal world, no child would experience extreme adversity, understanding the protective factors that promote resilient emotion regulation and healthy developmental outcomes following adversity is critical for addressing mental health problems globally (6).

To date, most resilience research has focused on high-income countries (HICs), a phenomenon known as the 10/90 divide, wherein only 10% of scientific knowledge is produced by or in low- and middle-income countries (LMICs)^a^ that comprise 90% of the world’s population (i.e., the majority world) (7, 8, 9, 10, 11, 12). However, children in LMICs are at high risk of failing to reach their developmental potential due to environmental and psychosocial risk factors (13, 14, 15). Therefore, studying diverse populations with a broad range of experiences is essential to advancing our understanding of resilience (16, 17, 18, 19). Assuming that research conducted in HICs is universal can impede scientific progress and recapitulate a colonial narrative of assuming developmental norms (20). This is not to invalidate research in HICs but rather to highlight that a more complete understanding of resilience must include other lived experiences. There is a rich literature on resilience in children and adolescents from LMICs (21, 22, 23, 24, 25, 26), but neurobiological research has lagged behind. Closing this research gap is an ethical imperative; characterizing flexible adaptation and neurodevelopmental change in children and adolescents from lower-resourced, high-adversity contexts will inform interventions that promote successful development in children and adolescents who are at the highest risk.

**Figure 1:**
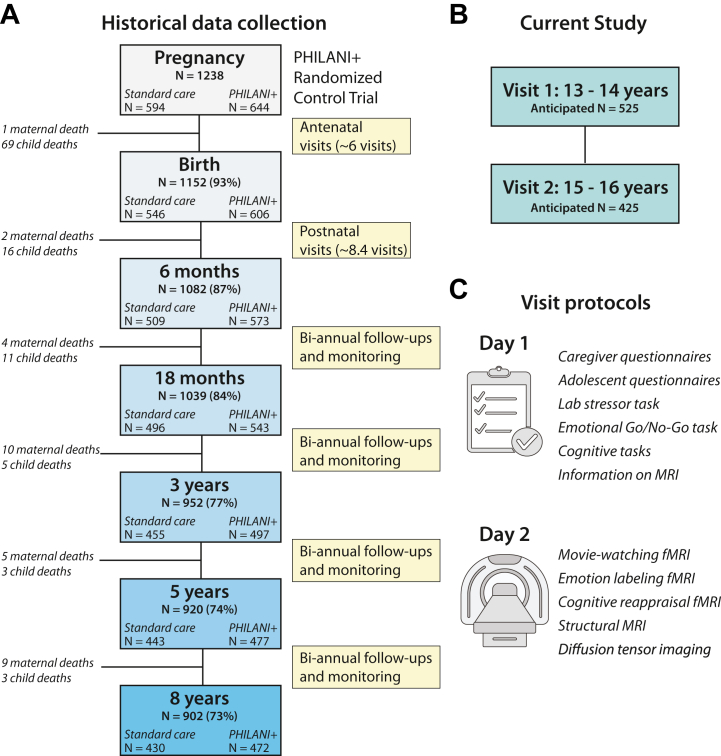
Study details. **(A)** Historical data collection timeline [modified from (28)]. Note that the numbers change according to loss to follow-up at various time points and subsequent return to the study. Thus, retention of the sample from the first time point is represented proportionally as percentages in parentheses. **(B)** Time points collected for the current study. **(C)** Protocol for data collection at both adolescent time points. fMRI, functional magnetic resonance imaging.

We have embarked on a longitudinal neurodevelopmental study of resilient emotion regulation in a low-income, high-adversity South African context (Figure 1) through a global research collaboration. We are re-enrolling 525 South African adolescents who have been tracked longitudinally from pregnancy (27,28). As detailed below, adolescents in this cohort were recruited from a township characterized by particularly high adversity. The cohort has experienced numerous adversities, starting with challenges faced by their mothers during pregnancy (e.g., infectious disease, alcohol use, depression, partner violence, and the intergenerational impacts of racism) (27,29) and continuing into the children’s own lives after birth. By definition, resilience is a phenomenon that can only be observed following exposure to adversity (16,18,19,30), meaning that the unfortunate, near-universal experience of adversity in the current sample may lead to important insights into the factors that contribute to positive developmental outcomes.

In this article, we outline an ongoing protocol for studying resilience and emotion regulation neurodevelopment in South African adolescents; our strategies for conducting methodologically robust, culturally relevant, and ethically sound research; the challenges and considerations in conducting this research; and the promise that this research holds both for the basic science of resilience and its clinical translation. We will make deidentified data from this project publicly available via the National Institute of Mental Health Data Archive. This article acts as a guide to the types of data being collected and provides context on the population and measures included.

### Resilient Emotion Regulation

Definitions of resilience vary, but the current scientific consensus is that it is not merely the opposite of vulnerability/risk or a stable trait; rather, resilience consists of a myriad of dynamic, protective factors that contribute to successful developmental outcomes following exposure to adversity (4,19,31, 32, 33). Resilience can include both processes that are internal to the individual, such as self-regulation (33), and factors that are external to the individual, such as social support in the form of caregivers, teachers, mentors, and friends (34) and resources in the community (35). These factors are part of a reciprocal system in which protective factors operate dynamically at multiple proximal and distal levels (36, 37, 38, 39). Systems of resilience have been investigated across various cultures, with caregivers playing a central role (22), particularly in establishing children’s adaptive socioemotional skills (40, 41, 42) and influencing the neurobiology that underlies emotion regulation (43, 44, 45) and associated resilience (31,46).

We focus on resilient emotion regulation in this project. Despite differences in beliefs about and desires for certain emotions across cultures (47, 48, 49, 50, 51, 52), having weak regulation over emotions is maladaptive (53, 54, 55). In South African adolescents, emotion dysregulation and poor coping strategies have been linked to internalizing symptoms and drug use (56,57). Cognitive reappraisal, in which a person thinks about a situation in a different way to change its emotional intensity or valence, is widely recognized as an adaptive emotion regulation strategy (58)—an effect that is seen across countries (59). Nonetheless, the paucity of research on emotion regulation in LMIC contexts means that questions remain about what adaptive (or maladaptive) emotion regulation strategies look like, the factors that predict their emergence, and how they are related to positive outcomes (60).

While it is unlikely that any one resilience factor is specific to one group, universal mechanisms of resilience (e.g., access to resources, social support, community affiliation, feelings of agency) will be informed by both culture and context (61,62). In other words, what makes an individual resilient in an HIC may not necessarily apply to an adolescent who is growing up in a peri-urban settlement in South Africa (35,63). For example, South African young people have described the cultural tradition of ubuntu—that is, showing “humanity to others”—as a key value important to developmental success (24). Ubuntu highlights community connectedness, spirituality, and cultural beliefs and has been identified as a source of resilience (26). Overall, it is important that resilience measures are culturally relevant and adapted before being used (64), as in the current research project.

In HICs, individual differences in emotion regulation are supported by brain networks that include the prefrontal cortex and limbic regions (i.e., amygdala) (65, 66, 67, 68), with activity in these regions linked to resilience (69). However, the functional development of the brain mechanisms associated with resilient emotion regulation have not been studied in LMIC contexts. Whether the same neurobiology supports resilient emotion regulation universally is an open question.

### Adolescence: A Neurobiological and Sociocultural Sensitive Period

Although emotion regulation is important across development, it is particularly salient during adolescence, an important transition period for emotion regulation and related neurobiology (70, 71, 72, 73). Adolescents transition from intrapersonal to independent emotion regulation strategies to successfully adapt to the changes that mark this developmental period (74,75). At the same time that these emotion regulation strategies are maturing, adolescence is marked by heightened sensitivity to the extrafamilial social environment, emotional reactivity, impulsivity, and risk taking (71,73,76,77). Perhaps reflecting this, caregivers from South Africa were most likely to report that early adolescence (10–15 years) is the most important time for caregiver support and intervention, citing the need to develop morals, self-protection, and life skills (78). Theoretical and empirical work (in HIC contexts) has also shown that adolescence is a biological sensitive period for the structural and functional development of neural systems relevant for affective processing, including emotion regulation (71,73,79, 80, 81, 82, 83). In sum, changes in emotion regulation and related affective processes during adolescence make it a period during which people are especially vulnerable to the emergence of mental health issues (84,85) and important for examining neurobiological mechanisms of resilient emotion regulation.

### The Current Project

**Figure 2:**
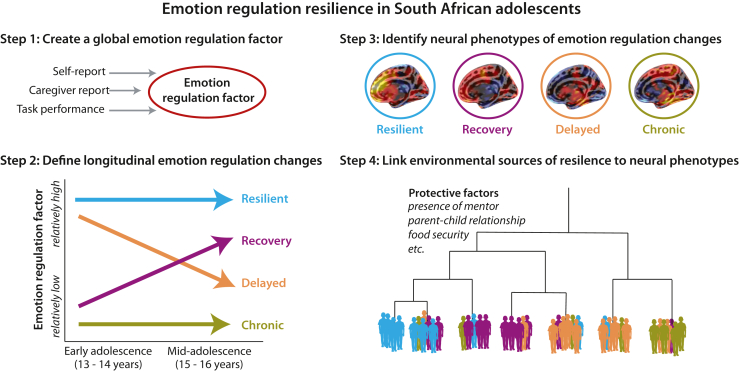
Analysis strategy for examining neurodevelopmental change profiles of resilient emotion regulation in South African adolescents. We take an exploratory, data-driven, and multilevel dimensional approach to overcome known limitations of single measures and to minimize assumptions made about resilient emotion regulation in adolescents from a low- and middle-income country (LMIC) context (see Table S6). There are 4 major parts to our analysis approach. First, we create a global emotion regulation factor informed by multiple methods (self-report, caregiver report, and task performance). Second, adolescents are identified as exhibiting resilient emotion regulation (or other developmental changes) based on their emotion regulation factor score across 2 time points. These canonical change profiles (resilient, recovery, delayed, and chronic) are informed by previous work that examined resilient change profiles following adversity. Third, neural phenotypes of resilient emotion regulation are identified using multiple sources of data (i.e., structural and functional). Finally, environmental sources of resilience (protective factors) are used to group participants and examine the link between protective factors and neural phenotypes of resilient emotion regulation. Caregiver-reported gender, age, and other covariates are considered across analyses, as are adversity profiles. Note that these images are for illustrative purposes only and do not reflect the data.

The primary aim of this project is to identify pathways to resilient emotion regulation and its neurobiological correlates during adolescence following adversity in an established South African birth cohort. As a transdiagnostic predictor of psychopathology (86, 87, 88), emotion regulation may be particularly useful in capturing the heterogeneity of mental health problems related to adversity exposure (89). By employing an exploratory, data-driven, and multilevel dimensional approach (including historical data, caregiver-/self-reports, performance measures, and longitudinal changes in magnetic resonance imaging [MRI] structure, connectivity, and task-based functional activity) (see Figure 2), this research will overcome some of the known limitations of categorical diagnoses as defined in the DSM (90,91). Additionally, we aim to reveal whether existing models of resilient emotion regulation generalize to adolescents from LMICs such as South Africa. Importantly, the measures used in this study were culturally adapted to the South African context through the input of focus groups and community members who serve as data collectors and key members of the research team. By including neuroimaging as a key component, this research will characterize neurodevelopmental change profiles in South African adolescents, thereby contributing to the growing field of neuroscience in Africa (92, 93, 94). Finally, understanding how aspects of the social and physical environment can serve as protective factors in adolescents from LMICs will advance preventive efforts and interventions.

## Methods and Materials

### Participants: The Philani Longitudinal Birth Cohort

Our study follows a longitudinal birth cohort of adolescents born in the Khayelitsha township in Cape Town, South Africa (27,95), an extremely low-income and high-adversity setting (see Supplemental Text). Pregnant women (*N* = 1238) were originally enrolled in a longitudinal randomized controlled trial to evaluate the effectiveness of a community-based home visiting program (see Figure 1A). Participants were divided into intervention groups based on the neighborhood that they lived in (*n* = 12 each), such that demographic factors were similar across groups. In the intervention group, local women, defined as peer influencers or mentor mothers, made home visits to support maternal and child nutrition in their geographic area. This original randomized controlled trial (from 2009–2011) was developed with Philani, a nongovernmental organization (96), to integrate the existing nutrition intervention with additional content and activities to address maternal health risks such as HIV, tuberculosis, nutrition, mental health, alcohol use, and healthy daily routines for mothers (27).

Results of the original randomized controlled trial and follow-ups have been published previously (28,29,95,97, 98, 99) (see Supplemental Text for a brief summary of the results). Given the effects of adversity observed in this sample, with or without the original intervention, there is value in following this cohort into adolescence to identify protective factors that promote individual differences in resilient emotion regulation and its neurobiology. This will be the first time that neuroimaging data have been collected with this cohort, meaning that the Philani sample will be the only LMIC birth cohort with a combination of high-density profiling of significant adversity/disparities (cataloged from the prenatal period to 8 years) together with neurobiological and behavioral phenotyping during adolescence.

We are recruiting 525 participants with the aim of retaining 425 adolescents across 2 visits occurring at 13 to 14 years of age (early adolescence) and 15 to 16 years of age (mid-adolescence) (Figure 1B). The pacing of the visits across 2 years was chosen to permit observation of developmental change while also being short enough to retain the sample. Our anticipated retention rate of 81% over 2 time points is a conservative estimate given the high rates of retention of this sample over the past decade (which ranges from 92%–98% across subsequent visits) (see Figure 1A). Recruitment is conducted by data collectors from the community who have longstanding relationships with the participants. To maintain a tight age range, participants >15 years of age at the time of the first visit are not enrolled. Because some participants might have migrated between the Western and Eastern Cape provinces (100), recruitment targets both locations. Exclusion criteria include significant neonatal medical complications, autism or significant intellectual disabilities, and MRI contraindications. Maternal HIV+ status during pregnancy is not considered an exclusion criterion given that vertical transmission rates have decreased considerably in South Africa due to improved access to antiretroviral therapy (101), which is reflected in the low incidence rates found in this cohort (2.4% of children with HIV at age 3 years) (102). Moreover, including adolescents born to mothers who are HIV+ is important to ensure the generalizability of our results to the study population. Nonetheless, maternal HIV status and known adolescent HIV status will be considered as covariates in follow-up robustness analyses to assess whether this adds additional variance to our results.

All participants are supported with fair compensation including meals and travel to and from study sites. We have a detailed neuroimaging incidental findings protocol and a referral system in place for participants who are in active distress or who need additional mental or physical health evaluation. Finally, the research is conducted by trained data collectors from the community who are fluent in isiXhosa and skilled in communicating with participants. This study was approved by the ethics committees at Stellenbosch University (HREC # N23/03/012) and Columbia University (IRB #AAAU5623).

### Aiming for Cultural Relevance

**Figure 3:**
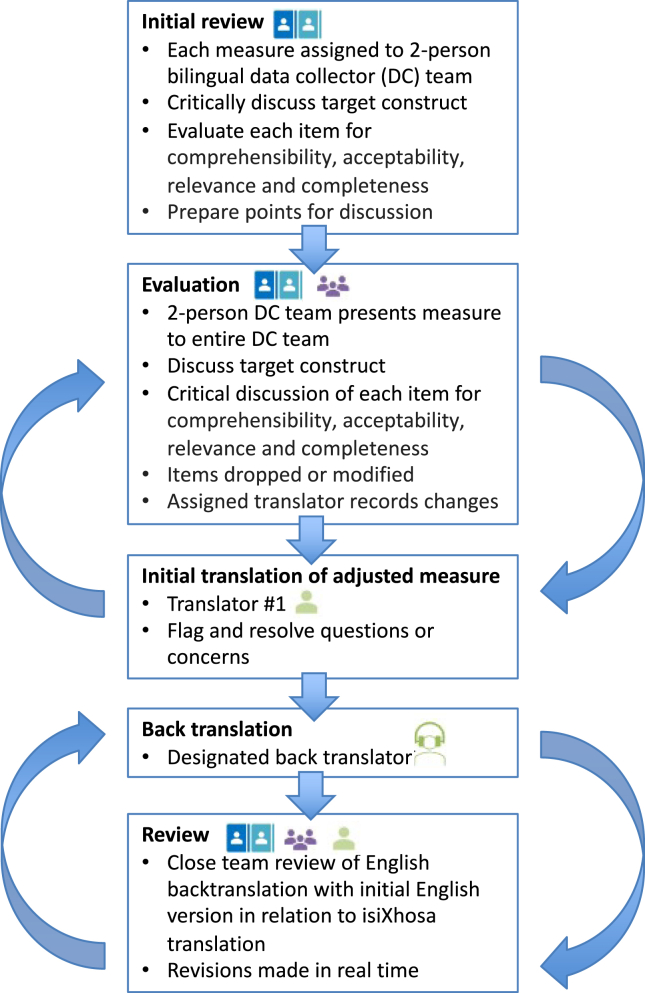
Cultural adaptation process for assessments used in the study. The procedure for cultural adaptation was iterative and consisted of translation, comprehension checks, back translation, and review.

Cultural adaptation is an essential practice in adapting psychosocial measures across diverse settings (64). Key to this is an understanding of the context of the target population. This includes community views, beliefs, values, and practices related to specific research questions (e.g., stress, coping mechanisms, child development) (103,104). The process of cultural adaptation used in the current study was guided by input from adolescent and adult community advisory boards and conducted by members of the research team who have expertise in cultural adaptation and/or have lived and worked in the community (see Positionality Statement) (78,105, 106, 107, 108). Adaptation was a rigorous and iterative process of translation, consultation, review, and back translation (outlined in Figure 3). The Supplemental Text summarizes the cultural adaptation process for this study; the outcomes of this process, including community perceptions and guidance, will be detailed in a forthcoming article.

### Behavioral and Neuroimaging Measures

Adolescents and their caregivers participate in a 2 day study visit twice across 2 years. On day 1, adolescents and their caregivers complete questionnaires and assessments at a research site in Khayelitsha. During this visit, prior to giving consent/assent, caregivers also receive MRI education through slideshows and videos presented in a standardized format and are given the opportunity to ask any questions. On day 2, adolescents receive the same MRI education and practice the MRI tasks prior to the MRI session (Figure 1C), which takes place at a nearby Cape Town hospital with a dedicated 3T research scanner. The MRI session lasts approximately 60 minutes, during which time data collectors from the community are in constant communication with adolescents to ensure participant comfort. The follow-up visit 2 years later follows the same behavioral and neuroimaging protocol to assess neurodevelopmental change profiles: resilient (high emotion regulation over time), recovery (initially low emotion regulation that increases), delayed (initially high or moderate emotion regulation that decreases), and chronic (low emotion regulation over time) (109,110). The assessment battery is summarized in Tables S1–S5 and detailed below. Assessments were chosen based on their strong psychometric properties, appropriateness for this sample given their previous use in the sample, and the outcomes of the cultural adaptation process.

Our primary aim is to examine emotion regulation. Previous work has found that emotion regulation during adolescence can be related to physical health and cognitive abilities (111,112). Therefore, we are collecting additional physical health and cognitive measures that have been validated in adolescent populations [e.g., (113)] to be used as covariates in our analyses (Table S1).

#### Measures of Adverse Experiences and Protective Factors

Adverse experiences from across the adolescents’ lifespan are measured through adolescent and caregiver reports (Table S2), as well as previously collected longitudinal data. Briefly, using standardized questionnaires and additional assessments, caregivers and adolescents report on their physical environment (e.g., where they live, neighborhood characteristics, access to food, predictability in the environment, access to the internet), negative life experiences (e.g., trauma, relationship violence, abuse and neglect, racial discrimination), and mental health (e.g., depression, anxiety, posttraumatic stress disorder, substance use). Measures were selected to complement historical data on adversity exposure in this sample. Additional assessments measure potential protective factors that may contribute to adolescent resilience (Table S3). Given that protective factors that contribute to adolescent resilience have not yet been characterized in this sample, we are assessing multiple possible sources of resilience including those raised in focus groups. Caregivers and adolescents are asked about the caregiver-child relationship, extrafamilial relationships (friendships, relationships with teachers, mentors), spiritual beliefs, feelings of connectedness to family and culture, daily activities and routines, goals and future-oriented thinking, and perceived social support. Condition in the previous randomized controlled trial (i.e., intervention or standard care group) is also considered as a potential protective factor.

#### Measures of Emotion Regulation and Resilience

Our primary outcome of adolescent resilient emotion regulation comprises multiple behavioral, caregiver, and self-report measures (Table S4). The rationale for assessing emotion regulation using a multilevel dimensional approach is to find a common higher-order factor of emotion regulation that is then related to neural phenotypes and protective factors. Caregivers and adolescents are asked about adolescents’ emotion reactivity and regulation, coping, social behavior, impulse control, goal-directed behavior, and motivation to regulate emotions. Adolescents’ physiological (i.e., skin conductance) and behavioral ratings of the intensity and valence of looming auditory stimuli (114) are measured to assess responses to stressors. An emotional faces Go/NoGo task (115) is used to assess cognitive control in the context of emotional information.

Implicit and explicit emotion regulation abilities are assessed using behavioral tasks acquired during functional MRI (fMRI). These tasks were chosen based on their previous use in behavioral and neuroimaging studies of emotion regulation. Although task-based fMRI has recently been shown to exhibit moderate to low test-retest reliability (116,117), including for measures of emotion processing (118, 119, 120), task-based fMRI provides high validity for our aim of investigating neurobiology relevant to emotion regulation and thus is an essential aspect of the research (121). An affect labeling task (122) is used to assess implicit emotion regulation, requiring participants either to choose the word that describes a facial expression (affect labeling condition) or a face of another person that matches the facial expression (affect matching condition). Behavioral (i.e., accuracy and response time) and neural differences between these conditions suggest differences due to the process of “putting emotions into words,” which is considered an implicit form of emotion regulation (123). To control for emotion reactivity, adolescents also perform a nonaffective matching condition (shape matching condition). All facial images shown are of Black people from the NimStim (124), following previous South African research (125). For explicit emotion regulation, adolescents perform a task used to assess the regulation of negative emotions via cognitive reappraisal, which in research in HICs is considered an adaptive regulation strategy (126). While viewing negative pictures from the International Affective Picture System (127) and the South African Affective Picture System (128), adolescents are instructed to try either to reduce their negative emotion by reappraising the picture’s content (reappraisal condition) or to look at the picture without trying to reduce negative feelings (attend-to-negative condition). Adolescents are also instructed to look at neutral pictures (attend-to-neutral condition). Neural differences between the attend-to-neutral and attend-to-negative conditions reflect emotional reactivity, while differences between the reappraisal and attend-to-negative conditions reflect the exertion of emotion regulation strategies via cognitive reappraisal. Adolescents undergo prescan training on cognitive reappraisal strategies to ensure that they understand the instructions and also answer postscan questions to assess the specific strategies that they used during scanning.

Finally, we collect structural and functional neuroimaging measures (Table S5) to identify neural phenotypes of resilient emotion regulation. Neuroimaging data can reveal whether the same neural circuits that underlie resilient emotion regulation in HIC contexts apply to this sample. The neuroimaging procedures and scanning parameters were chosen to yield valid neuroimaging variables previously connected to emotion regulation, minimize participant burden, and roughly harmonize with imaging acquisition from a large longitudinal study of adolescents in the United States [the Adolescent Brain Cognitive Development (ABCD) Study (129)]. The use of multiband fMRI acquisitions enables higher spatial resolution for imaging subcortical structures (e.g., amygdala), examining fine-grain representations, and conducting surface-based analyses for functional connectivity (130). Task-based fMRI during implicit and explicit emotion regulation tasks (described above) are collected to examine functional neural activity during emotion regulation processes. Structural scans (T1-weighted) are collected to measure cortical volume and thickness across the brain and in particular regions of interest (e.g., the prefrontal cortex) (131). Diffusion tensor imaging scans are collected to measure structural connectivity between brain regions, such as between the prefrontal cortex and the amygdala (132), and to investigate the maturation of white matter microstructure, which exhibits well-documented changes during adolescence (133,134). To measure functional connectivity between regions, adolescents watch 2 short movie clips (“The Present” and “Homeward Bound”) during a resting-state fMRI scan (135,136). Both movies are narratives that have been used in previous developmental neuroimaging research and include socioemotional content but differ in the extent to which caregiver attachment relations are evoked, enabling exploratory analyses of naturalistic socioemotional stimulus processing (137). The movie-watching resting state occurs at the start of the MRI session, following the structural scan, to reduce the impact of posttask effects on functional connectivity (138). Next, participants complete the emotion regulation fMRI tasks and the diffusion tensor imaging scan. Finally, an additional functional scan is collected at the end of the session to infer eye gaze during neuroimaging data collection using a predictive modeling approach (139).

### Considerations for Analyses

The rich, multimodal, and longitudinal nature of this project creates the opportunity for us to answer a number of research questions about resilient emotion regulation neurodevelopment in an LMIC context. Moreover, because we plan to make this data publicly available, it is important to provide context and considerations for future data analyses. We have specifically chosen not to collect data from a comparison group outside of this context. Cross-cultural comparisons, if not done properly, can inadvertently lead to further marginalization of at-risk and minority groups (140). Thus, it is important to maintain the cultural context in which this research is being conducted. As we mentioned previously, adversity is nearly universal in the community in which the participants live, meaning that there would be no suitable no-adversity comparison group within the cultural context of this research. Instead, our primary aim in this study is to examine individual differences and pathways to emotion regulation resilience within the Philani longitudinal birth cohort; doing so allows us to move away from a deficit-only perspective of adversity and toward research that promotes resilience in this population (141).

We also had to consider whether theories and constructs derived from HICs would be appropriate to the context of this study. Much of our understanding of resilience following adversity derives from populations that have experienced different types and magnitudes of adversity than the adolescents in the current study. Thus, we opted to use data-driven approaches to characterize adversity and emotion regulation resilience in this population (89) (Figure 2). For example, rather than grouping adolescents by hypothesized dimensions of adversity [e.g., threat and deprivation, harshness and unpredictability (142,143)], we will use a clustering approach to create adversity subgroups specific to this population (see Table S6 for the analytic plan). Additionally, rather than relying on a single measure of emotion regulation, we opted to aggregate multiple types of data (i.e., survey responses, task behaviors) into a global emotion regulation factor that we will then relate to different protective factors. Data-driven analyses have been successfully used in previous neurobiological and behavioral phenotyping research (144, 145, 146, 147). For neural measures, while we will consider regions of interest that have previously been shown to be related to resilience in HIC contexts (e.g., the prefrontal cortex, amygdala), our primary analysis approach will be to characterize whole-brain neural phenotypes for resilience. From this methodology, we will uncover region and network contributions to resilient emotion regulation that may answer questions about the localization and laterality of emotion processing (148,149). This neural phenotyping approach has the additional advantage of mitigating concerns over signal to noise for any given region/task that may be due to task reliability (116,117) or the use of multiband acquisitions for fMRI (150,151). All analyses will consider relative differences across measures within the study population, rather than using external benchmarks, to again maintain the cultural context and minimize assumptions.

## Discussion

Many children and adolescents in LMICs experience profound adversities, but these samples have been underrepresented in research on neurobiological mechanisms of resilience. Here, we described our strategies for investigating the neurodevelopmental mechanisms that underlie resilient emotion regulation in a South African cohort. We have emphasized the need to understand resilience and neurodevelopmental change profiles in the context and culture of this sample to reduce the 10/90 research divide (7,8,10) and provide insights into resilience, emotion regulation, and neurodevelopment in adolescents from an LMIC.

The current work has limitations that should be considered. First, although we are leveraging dense historical data from across adolescents’ lives, the original study was not designed to characterize resilience. Thus, we do not have the ability to consider how early protective factors (i.e., during infancy and childhood) are related to current resilient emotion regulation. Future work is needed to advance our understanding of sensitive periods for resilience (152). Second, the final planned sample size for neuroimaging data collection will be moderate compared with other large-scale consortium studies (anticipated *N* = 525 at time point 1 and *n* = 425 at time point 2). There have been several recent debates over the appropriate sample size for relating brain and behavior, with some analyses requiring thousands of individuals (153, 154, 155, 156, 157). Our approach of collecting rich, phenotypic data from individual participants; using high signal-to-noise, task-based fMRI; and using machine learning methods with cross-validation techniques mitigates some of these concerns. Another strength of our approach is going beyond cross-sectional individual differences to examine how protective factors predict change profiles over 2 time points within the early adolescence to mid-adolescence period. Previous research has shown that measurable change can be acquired with few time points in the adolescent age range (158,159) and that this approach is statistically valid over longer study durations (160). However, it is possible that not all of the canonical change profiles (resilient, recovery, delayed, chronic) (Figure 2) are observable in this sample. In this event, we will adopt machine learning–based clustering methods to uncover change profiles in a data-driven way (see Table S6). Additionally, although we anticipate high retention given historical success, attrition is always a challenge, and we will employ appropriate statistical measures if necessary to account for possible biases (161,162).

No child should have to bear the burden of developing resilience as a response to familial, structural, and/or health inequities. However, identifying modifiable sources of resilient emotion regulation that mitigate risk factors offers promise for promoting positive neurodevelopmental and mental health outcomes in individuals who are growing up with considerable adversity. By focusing on South African adolescents with extreme adversity, this research can inform new models of risk and resilience for adolescents from LMICs. Ultimately, our hope is that findings from this research will inform models for mental health around the world.

### Positionality Statement

We have prioritized a research approach that reflects a diverse set of voices and avoids replicating historical power hierarchies between countries. There is an unfortunate history of science being conducted by researchers from HICs on participants from low-income countries [sometimes referred to as helicopter or parachute research (10,163, 164, 165)]. Our approach has been to instead echo the principles of interconnectedness, collaboration, and mutual respect that are central to the ubuntu philosophy (166) and to conduct collaborative work that is both inclusive and culturally competent. From the outset, community members have participated in the research, not just as temporary consultants, but also as leading, valuable members of the research team (see Aiming for Cultural Relevance). We aim for an equitable global collaboration where information transfer is bidirectional and considers the expertise and perspectives of all team members. In line with these goals, the project researchers continuously learn from team members of various disciplines, both through formal training and knowledge sharing. In the current paper, 57.1% of the authors are working in the United States, and 42.9% of the authors are working in South Africa; 28.6% of authors have worked with the Philani longitudinal birth cohort since its inception in 2009. The authors include experts in psychology, developmental science, neuroscience, psychiatry, medicine, public health, anthropology, informatics, open science, and community-based research. Seventeen of the authors self-reported additional demographic information: 5.9% identify as Asian/White, 29.4% identify as Black African, 5.9% identify as Indian, 5.9% identify as Latin/White, 5.9% identify as Middle East/North African, and 47.1% identify as White/Caucasian^b^; 29.4% of authors speak isiXhosa, and 11.8% of authors speak another common South African language (e.g., Afrikaans).

## Supplementary Material

Supplement Text and Tables S1–S6

Key Resources Table

## Footnotes

a There are many different terms that can be used to characterize the types of countries that have been included in the majority of scientific research and those that have been excluded [e.g., HICs vs. LMICs, Global North vs. Global South, minority world vs. majority world (167)].

## Footnotes

b Note that self-identified race/ethnicity categories can vary by country.
