# Supplementary material for: Investigative Approaches to Resilient Emotion Regulation Neurodevelopment in a South African Birth Cohort
Source: Biol Psychiatry Glob Open Sci. 2025 Jan 31;5(3):100457. doi: 10.1016/j.bpsgos.2025.100457 (PMC11938085; doi:10.1016/j.bpsgos.2025.100457)
Supplement: Supplement Text and Tables S1–S6 [file mmc1.pdf]

# SUPPLEMENTARY INFORMATION

# Investigative Approaches to Resilient Emotion Regulation Neurodevelopment in a South African Birth Cohort

Yates, Sigwebela, *et al.*

**Supplemental Text**

***Adversity in the Philani longitudinal birth cohort***

The Philani cohort has experienced a level of adversity that is rarely studied in resilience research conducted in HIC contexts. In South Africa, more than half of the population faces high rates of intersecting socio-economic challenges. In fact, child poverty in South Africa is among the highest globally, with nearly 62% of children aged 0-17 experiencing multidimensional poverty (1), and 75% of Black children living in income-poor households (2), hindering their psychosocial development (3). Other social determinants include environmental safety factors such as high levels of crime, substance misuse, and gender-based violence (4). Adversity exposure is particularly prominent in Khayelitsha (the recruitment location of the cohort), which is characterized by high levels of poverty and violence, with ~70% of residents living in informal housing (e.g., shacks), ~33% without access to a working toilet or clean water, and ~89% of households moderately or severely food insecure (5). Access to psychosocial services and health support is limited due to resource constraints such as an overburdened health care system, low levels of education, inequality, and slowly-developing infrastructure in both remote and peri-urban settings (6). While maternal and health services are available, these services do not typically address the socio-emotional needs of caregivers and their children and those that do are administratively overburdened (5).

***Previous results in the Philani longitudinal birth cohort***

At the time of data collection, about 26% of township mothers were living with HIV (5). Use of alcohol during pregnancy at the time of data collection was between 9-26% (7), and 17% of women had clinically significant levels of depressed mood (8). Only 19% of the women were employed at the time of data collection (7). Results of the original randomized controlled trial (RCT) and follow ups have been published previously (7–14). In brief, the intervention had significant effects on child outcomes, alcohol use, and maternal depression at the 18-month to 5-year follow-ups (7,8,10,11,14). Benefits from the intervention group were evident as early as 18 months, with positive effects observed in terms of infants' health and growth, particularly through increased breastfeeding. Prevalence of perinatal depression was significantly lower in the intervention group at the 36-month follow up. However, the effects of the intervention did not continue beyond early childhood, with no significant differences in child outcomes at the 5- and 8-year follow-ups (11,12). Importantly, analyses across both the intervention and control groups revealed the large impact of adversity on child development. For example, maternal depression affected mother-child interactions at 3 years (14) and problematic maternal alcohol use and depression increased children's aggression at 3 and 5 years (9,14).

***Cultural adaptation process***

For the current study, cultural adaptation took place in two phases: identifying key local issues and relevant measures, and the cultural adaptation of these measures. For part one, we followed recommendations for understanding contextual information about the target population (15–18). Specifically, seven focus groups were held to map the relevant cultural landscape, with the aim of identifying key issues and beliefs about adolescents, adolescent development, and parenting. These sessions were held alongside frequent consultations with key informant staff members who were longstanding members of the community. On the basis of this, we selected a battery of appropriate measures. In the adaptation phase, construct and content were critically evaluated by the bilingual data collection team and adjusted according to comprehensibility, acceptability, relevance, and completeness of each measure, then translated to isiXhosa followed by backtranslation. The process of translation and adaptation was a rigorous and iterative process of translation, consultation, review, and back translation by a team of designated translators and data collectors (outlined in **Figure 3)**. This iterative method helps

to ensure that the measures are conceptually, semantically, and functionally comparable (19–21). At completion, the final inventory of adapted measures, which includes both newly-adapted measures for the current study and measures previously adapted for this sample, was then piloted with 20 participants from the sample to understand how participants engage with the instrument and interpret the questions (22).

Community advisory boards can provide insight and guidance into research activities and improve the social and cultural relevance of research projects (23). Throughout the project development phase, adult and adolescent community advisory boards were consulted for guidance pertaining to the project messaging and assessment materials. Insights gained from the cultural adaptation process will be the subject of a forthcoming manuscript.

## Supplemental Tables

**Additional Physical Health and Cognitive Measures**

| Measure | Brief summary | Citation |
|---|---|---|
| *Adolescent Questionnaires/Assessments* | | |
| Child BMI | adolescent's height and weight | n/a |
| Peterson Pubertal Development Scale (PPDS)* | measure of pubertal status | (24) |
| Pittsburgh Sleep Quality Index (PSQI) | adolescent's sleeping habits | (25) |
| Two-choice reaction time task | computer task measuring reaction time and processing speed | (26) |
| Backwards digit span | computer task measuring working memory capacity | (27) |
| NIH toolbox - pattern comparison | computer task measuring pattern recognition and processing speed | (28) |
| NIH toolbox - flanker | computer task measuring inhibitory control and executive function | (28) |
| Spiral task | tracing task to measure fine-motor skills | n/a |
| Raven's progressive matrices | test of abstract reasoning and non-verbal fluid intelligence | (29) |

**Table S1.** List of additional questionnaires and assessments on adolescents' physical health and cognition. * = newly culturally adapted for the current study.

| Measures of Adverse Experiences | | |
|---|---|---|
| **Measure** | **Brief summary** | **Citation** |
| *Caregiver and Adolescent Questionnaires/Assessments* | | |
| Household Food Insecurity Access Scale (HFIAS) # | how often household eats food | (30) |
| MacArthur Scale of Subjective Social Status (SSS)* | perception of financial circumstances and socio-economic status | (31) |
| Index of Race-Related Stress (IRRS)* | experiences with racism | (32) |
| Substance use# | recent substance use (smoking, alcohol, other drugs) | n/a |
| *Caregiver Questionnaires/Assessments* | | |
| Demographic information about housing | housing situation and amenities (water, electricity, appliances) | n/a |
| Childcare and migration# | caregiver relation to child and travel to Eastern Cape | n/a |
| Income and employment# | caregiver employment, financial support, and household income | n/a |
| Relationship status# | caregiver relationship history and sexual history | n/a |
| Relationships and violence | any abuse (physical and verbal) caregivers experienced | n/a |
| Life Events Checklist 5* | any traumatic events or situations the caregiver has experienced | (33) |
| Maternal health | caregiver health status and where they go to get health support | n/a |
| PTSD Checklist 5 | assessment of PTSD symptoms in caregiver | (34) |
| PHQ-9# | assessment of depressive symptoms in caregiver | (35) |
| GAD-7# | assessment of anxiety symptoms in caregiver | (36) |
| *Adolescent Questionnaires/Assessments* | | |
| COVID | whether someone the adolescent knows passed away from COVID | n/a |
| PhenX: Neighborhood and community network | neighborhood safety and connections to the community | (37) |
| Questionnaire of Unpredictability in Childhood (QUIC)* | unpredictability in adolescent social support and home environment | (38) |
| Internet access | adolescent's access to the internet and what they use it for | n/a |
| CBCL: Rule breaking# | adolescent involvement in gang activity and crime | (39) |
| Child Trauma Questionnaire (CTQ) | history of maltreatment of the adolescent | (40) |
| Child Trauma UCLA Questionnaire | trauma exposure and any symptoms of PTSD in adolescent | (41) |
| The Child PTSD Symptom Scale (CPSS) | symptoms and intensity of PTSD in adolescent | (42) |
| Revised Children's Anxiety and Depression Scale (RCADS) | symptoms of anxiety and depression in adolescent | (43) |

**Table S2.** List of questionnaires and assessments used to measure adverse experiences in the adolescent. * = newly culturally adapted for the current study. # = Used at previous timepoint.

**Measures of Protective Factors**

| Measure | Brief summary | Citation |
|---|---|---|
| *Caregiver and Adolescent Questionnaires/Assessments* | | |
| Multidimensional Scale of Perceived Social Support (MSPSS) | opinion on getting support from family, friends or partner | (44) |
| *Caregiver Questionnaires/Assessments* | | |
| Maternal family# | information about child's mother and grandmother | n/a |
| Paternal family# | information about child's father and grandfather, and father involvement | n/a |
| Religiosity* | caregiver spiritual and/or religious beliefs | n/a |
| Parental Stress Scale (PSS) | caregiver experience of being a parent to their child | (45) |
| Alabama Parenting Questionnaire (APQ) | parenting habits, both positive and negative parenting practices | (46) |
| Correctors of child misbehavior | actions caregiver takes to address their child's behavior | (12) |
| Hospitalizations & clinic treatment# | location and quality of the medical care child receives | n/a |
| Routines | caregiver's daily activities and routines | n/a |
| School | caregiver's perception of child's school experience | n/a |
| Hope-future orientation | caregiver's hopes for their child's education and future life | (47) |
| State Hope Scale (SHS)* | caregiver's goals and how they are achieving them | (48) |
| *Adolescent Questionnaires/Assessments* | | |
| Parent IOS* | adolescent's feelings of closeness to their caregiver | n/a |
| Culture IOS* | adolescent's connectedness to family beliefs or traditional culture | n/a |
| Parent Child Relationship Scale (PCRS)* | adolescent's perception of caregiver warmth, positivity, and communication | (49) |
| Preoccupied and Avoidant Coping Questionnaire (PACQ)* | adolescent's feelings about how responsible and trustworthy their caregivers are | (50,51) |
| CBCL: Extracurricular activities* | any sports, societies, games, chores, and habits of the adolescent | (39) |
| Household responsibilities | adolescent's chores and support they provide in their home | n/a |
| Friendship* | adolescent's friends or friendship group | n/a |
| Benevolent childhood experiences (BCE) and Mentor questionnaire* | whether and how adolescent receive support from a mentor | (52,53) |
| Teacher as resource* | adolescent's relationship with a teacher as a supportive non-familial adult | n/a |
| Theories of intelligence and growth mindset* | adolescent's ideas about intelligence and learning capacity | (54) |

**Table S3.** List of questionnaires and assessments used to measure protective factors in adolescence. * = newly culturally adapted for the current study. # = Used at previous timepoint.

**Measures of Emotion Regulation and Resilience**

| Measure | Brief summary | Citation |
|---|---|---|
| *Caregiver and Adolescent Questionnaires/Assessments* | | |
| Difficulties in Emotion Regulation Scale-SF (DERS-SF) | how adolescent manages or controls negative emotions | (55) |
| Strengths and difficulties Questionnaire (SDQ) [#] | how adolescent considers, helps, and interacts with others | (56) |
| *Caregiver Questionnaires/Assessments* | | |
| Child behavior checklist (CBCL) [#] | caregiver report of adolescent's behavioral and emotional problems | (39) |
| SWAN rating scale | caregiver report of adolescent's ADHD symptoms, ability to focus, control impulses | (57) |
| Social Aptitude Scale (SAS) | caregiver's perception of adolescent's social skills compared to other children their age | (58) |
| Connor-Davidson Resilience Scale (CDRS) | caregiver's perception of how their child deals with challenges, adapts and copes with stress and difficult life experiences | (59) |
| *Adolescent Questionnaires/Assessments* | | |
| Coping Scale for Children and Youth (CSCY)* | adolescent's coping strategies including how they solve problems, ask for help and motivate themselves | (60) |
| Deliberate Self Harm Inventory (DSHI) | the frequency and duration the adolescent has engaged in self-harm | (61) |
| Children's Hope Scale* | adolescent's ability to set and maintain their goals | (62) |
| Resilience Scale* | how adolescent handles challenges | (63) |
| Importance of emotion regulation* | how adolescent expresses or controls their emotions and its importance to them | (64,65) |
| Laboratory-based stressor | computer task assessing adolescent behavioral and physiological responses to looming sounds | (66) |
| Emotional face go/no-go task* | computer task assessing how adolescents respond and inhibit responses when viewing emotional face stimuli | (67) |
| Affect labelling fMRI task* | computer task during functional MRI measuring implicit emotion regulation | (68) |
| Cognitive reappraisal fMRI task* | computer task during functional MRI measuring explicit emotion regulation | (69) |
| Movie-watching fMRI task | passive viewing of emotional movies during functional MRI, measuring brain responses and functional connectivity | (70,71) |
| Cortical Volume/Thickness | measure of cortical volume/thickness in structural MRI scans | n/a |
| Structural connectivity | measure of structural connectivity in diffusion tensor imaging MRI scans | n/a |
| White matter microstructure | measure of tissue microstructure (fiber integrity, orientation) in diffusion tensor imaging MRI scans | n/a |

**Table S4.** List of questionnaires and assessments used to measure resilience and emotion regulation in the adolescent. * = newly culturally adapted for the current study. # = Used at previous timepoint.

| Sequence | Slices | % FOV phase | Resolution (mm) | TR (ms) | TE (ms) | TI (ms) | Flip Angle (°) | Multi Band Accel | Phase Partial Fourier | Notes |
|---|---|---|---|---|---|---|---|---|---|---|
| T1 MPRAGE | 176 | 100% | 1.0x1.0x1.0 | 2530 | 1.68 | 1240 | 7 | N/A | Off | |
| Diffusion | 84 | 100% | 1.7x1.7x1.7 | 3690 | 111.8 | N/A | 78 | 4 | 6/8 | 104 directions, b=0.2000 |
| fMRI | 60 | 100% | 2.4x2.4x2.4 | 832 | 34.6 | N/A | 52 | 6 | Off | |

**Table S5.** MRI parameters used in the current study. Data are collected at Cape Universities Body Imaging Center (CUBIC) with a Siemens 3T Skyra scanner dedicated for research purposes.

| Planned analyses for examining emotion regulation resilience in South African adolescents | |
|---|---|
| **Analysis step** | **Analytic plan** |
| Adversity subtyping | Create reproducible and generalizable clusters of participants based on adversity factors from across adolescents' lifetime using machine learning approaches (e.g., Bagging-Enhanced Factor Analysis ($EFA_{be}$) with cross-validation and Louvain Community Detection ($LCD_{be}$) (72) |
| Emotion regulation resilience factor in early adolescence (timepoint 1) | Create a global emotion regulation factor from multiple methods (caregiver/self-report and behavioral performance measures) for broad coverage of emotion regulation abilities using machine learning ($EFA_{be)}$ (73) |
| Longitudinal changes in emotion regulation resilience (timepoints 1 and 2) | Examine changes in emotion regulation resilience over adolescence by applying factor structure obtained using timepoint 1 data on timepoint 2 data, then uncover subtypes of emotion regulation resilience change using $LCD_{be}$ clustering framework and/or canonical change subtypes (resilient, recovery, delayed, chronic) (74,75) |
| Structural MRI preprocessing | Obtain measures of brain structure (e.g., grey matter volume, cortical thickness) following preprocessing (e.g., bias field correction, anatomical registration to MNI-152 standard space and Mindboggle for anatomical labeling) (76) |
| Diffusion Tensor Imaging (DTI) preprocessing | Obtain measures of structural connectivity following preprocessing (e.g., motion and eddy current distortion correction, probabilistic fiber tracking algorithms) (77) |
| Functional MRI preprocessing | Obtain measures of brain function (e.g., task activation, global functional connectivity) following preprocessing (e.g., distortion correction, temporal denoising, motion correction, anatomical registration to MNI-152 standard space, nuisance signal regression) using Configurable Pipeline for the Analysis of Connectomes (C-PAC) (78) |
| Neural phenotypes of emotion regulation resilience and change | Uncover the neural correlates of emotion regulation resilience and its longitudinal change over adolescence using an analysis of covariance (ANCOVA) including interactions with adversity subtype and age/sex covariates. Analyses done in *a priori* regions and networks of interest (e.g., amygdala, hippocampus, medial prefrontal cortex, lateral prefrontal cortex, 7 Yeo Atlas networks) and in an exploratory voxel-wise analysis with threshold free cluster enhancement for multiple comparisons correction (79) |
| Predictive model of emotion regulation resilience change based on environmental exposures | Determine the influence of adverse and protective factors on longitudinal measures of emotion regulation resilience and its neural correlates using an unbiased and predictive machine learning technique (e.g., conditional random forests with cross-validation) (80) |

**Table S6.** Examples of planned analyses for examining longitudinal changes in emotion regulation resilience, neural phenotypes, and the influence of adverse experiences and protective factors in South African adolescents exposed to adversity. Rigorous exploratory, data-driven analyses are prioritized given that little prior research has considered resilience in adolescents from South Africa, leaving open the possibility that theories and constructs developed in high-income countries may not apply.

**Supplemental References**


1. Statistics South Africa (2020, July 7): South Africa's poor little children. Retrieved May 22, 2024, from https://www.statssa.gov.za/?p=13422
2. Hall K (2024, March): Children Count. Retrieved June 18, 2024, from http://childrencount.uct.ac.za/indicator.php?domain=2&indicator=98
3. Statistics South Africa (2024, May 22): People of South Africa. Retrieved May 22, 2024, from https://www.gov.za/about-sa/people-south-africa-0
4. Christodoulou J, Rotheram-Borus MJ, Hayati Rezvan P, Comulada WS, Stewart J, Almirol E, Tomlinson M (2022): Where you live matters: Township neighborhood factors important to resilience among south African children from birth to 5 years of age. *Preventive Medicine* 157: 106966.
5. Rotheram-Borus MJ, le Roux IM, Tomlinson M, Mbewu N, Comulada WS, le Roux K, *et al.* (2011): Philani Plus (+): A Mentor Mother Community Health Worker Home Visiting Program to Improve Maternal and Infants' Outcomes. *Prevention Science* 12: 372–388.
6. Burger R, Christian C (2020): Access to health care in post-apartheid South Africa: availability, affordability, acceptability. *Health Economics, Policy and Law* 15: 43–55.
7. Le Roux IM, Rotheram-Borus MJ, Stein J, Tomlinson M (2014): The impact of paraprofessional home visitors on infants' growth and health at 18 months. *Vulnerable Children and Youth Studies* 9: 291–304.
8. Tomlinson M, Rotheram-Borus MJ, Scheffler A, Roux I le (2018): Antenatal depressed mood and child cognitive and physical growth at 18-months in South Africa: a cluster randomised controlled trial of home visiting by community health workers. *Epidemiology and Psychiatric Sciences* 27: 601–610.
9. Gordon S, Rotheram-Fuller E, Rezvan P, Stewart J, Christodoulou J, Tomlinson M (2021): Maternal depressed mood and child development over the first five years of life in South Africa. *Journal of Affective Disorders* 294: 346–356.
10. Le Roux IM, Tomlinson M, Harwood JM, O'Connor MJ, Worthman CM, Mbewu N, *et al.* (2013): Outcomes of home visits for pregnant mothers and their infants: a cluster randomized controlled trial. *Aids* 27: 1461–1471.
11. Rotheram-Borus MJ, Tomlinson M, Roux IL, Stein JA (2015): Alcohol Use, Partner Violence, and Depression: A Cluster Randomized Controlled Trial Among Urban South African Mothers Over 3 Years. *American Journal of Preventive Medicine* 49: 715–725.
12. Rotheram-Borus MJ, Tomlinson M, Worthman CM, Norwood P, le Roux I, O'Connor MJ (2023): Maternal depression, alcohol use, and transient effects of perinatal paraprofessional home visiting in South Africa: Eight-year follow-up of a cluster randomized controlled trial. *Social Science & Medicine* 324: 115853.
13. Tomlinson M, Rotheram-Borus MJ, Harwood J, Le Roux IM, O'Connor M, Worthman C (2015): Community health workers can improve child growth of antenatally-depressed, South African mothers: a cluster randomized controlled trial. *BMC Psychiatry* 15: 225.
14. Tomlinson M, Hartley M, Le Roux IM, Rotheram-Borus MJ (2016): The Philani Mentor Mothers Intervention: neighbourhood wide impact on child growth in Cape Town's peri-urban settlements. *Vulnerable Children and Youth Studies* 11: 211–220.
15. Baradaran Eftekhari M, Mahmoodi Z, Dejman M, Forouzan AS, Falahat K, Shati M, *et al.* (2021): Local perceptions of mental health in Iran, Semnan Province. *Brain and Behavior* 11: e01971.
16. Holstein JA, Gubrium JF (1995): *The Active Interview*, vol. vii. Sage Publications, Inc.
17. Ndambo MK, Munyaneza F, Aron MB, Nhlema B, Connolly E (2022): Qualitative assessment of community health workers' perspective on their motivation in community-based primary health care in rural Malawi. *BMC Health Services Research* 22: 179.

18. Snodgrass JG, Lacy MG, Upadhyay C (2017): Developing culturally sensitive affect scales for global mental health research and practice: Emotional balance, not named syndromes, in Indian Adivasi subjective well-being. *Social Science & Medicine* 187: 174–183.
19. Flaherty JA, Gaviria FM, Pathak D, Mitchell T, Wintrob R, Richman JA, Birz S (1988): Developing instruments for cross-cultural psychiatric research. *The Journal of Nervous and Mental Disease* 176: 260–263.
20. Kohrt BA, Jordans MJ, Tol WA, Luitel NP, Maharjan SM, Upadhaya N (2011): Validation of cross-cultural child mental health and psychosocial research instruments: adapting the Depression Self-Rating Scale and Child PTSD Symptom Scale in Nepal. *BMC Psychiatry* 11: 127.
21. Van Ommeren M, Sharma B, Thapa S, Makaju R, Prasain D, Bhattarai R, De Jong J (1999): Preparing Instruments for Transcultural Research: Use of the Translation Monitoring Form with Nepali-Speaking Bhutanese Refugees. *Transcultural Psychiatry* 36: 285–301.
22. Willis GB (2004): *Cognitive Interviewing: A Tool for Improving Questionnaire Design*. Sage Publications, Inc.
23. Yuan NP, Mayer BM, Joshweseoma L, Clichee D, Teufel-Shone NI (2020): Development of Guidelines to Improve the Effectiveness of Community Advisory Boards in Health Research. *Progress in Community Health Partnerships* 14: 259–269.
24. Petersen AC, Crockett L, Richards M, Boxer A (1988): A self-report measure of pubertal status: Reliability, validity, and initial norms. *Journal of Youth and Adolescence* 17: 117–133.
25. Buysse DJ, Reynolds CF, Monk TH, Berman SR, Kupfer DJ (1989): The Pittsburgh sleep quality index: A new instrument for psychiatric practice and research. *Psychiatry Research* 28: 193–213.
26. Simioni AR, Pine DS, Sato JR, Pan PM, Fonseca RP, Schafer J, *et al.* (2019, November 27): A Cognitive Development Chart for School-age Children and Adolescents. medRxiv, p 19012963.
27. Woods DL, Kishiyama MM, Yund EW, Herron TJ, Edwards B, Poliva O, *et al.* (2011): Improving digit span assessment of short-term verbal memory. *Journal of Clinical and Experimental Neuropsychology* 33: 101–111.
28. Weintraub S, Dikmen SS, Heaton RK, Tulsky DS, Zelazo PD, Bauer PJ, *et al.* (2013): Cognition assessment using the NIH Toolbox. *Neurology* 80: S54–S64.
29. Raven J (2000): The Raven's Progressive Matrices: Change and Stability over Culture and Time. *Cognitive Psychology* 41: 1–48.
30. Coates J, Swindale A, Bilinsky P (2007): Household Food Insecurity Access Scale (HFIAS) for Measurement of Food Access: Indicator Guide: Version 3: (576842013-001).
31. Goodman E, Adler NE, Kawachi I, Frazier AL, Huang B, Colditz GA (2001): Adolescents' Perceptions of Social Status: Development and Evaluation of a New Indicator. *Pediatrics* 108: e31.
32. Utsey SO, Ponterotto JG (1996): Development and validation of the Index of Race-Related Stress (IRRS). *Journal of Counseling Psychology* 43: 490–501.
33. Gray MJ, Litz BT, Hsu JL, Lombardo TW (2004): Psychometric Properties of the Life Events Checklist. *Assessment* 11: 330–341.
34. Blevins CA, Weathers FW, Davis MT, Witte TK, Domino JL (2015): The Posttraumatic Stress Disorder Checklist for DSM-5 (PCL-5): Development and Initial Psychometric Evaluation. *Journal of Traumatic Stress* 28: 489–498.
35. Kroenke K, Spitzer RL, Williams JBW (2001): The PHQ-9. *Journal of General Internal Medicine* 16: 606–613.
36. Spitzer RL, Kroenke K, Williams JBW, Löwe B (2006): A Brief Measure for Assessing Generalized Anxiety Disorder: The GAD-7. *Archives of Internal Medicine* 166: 1092–1097.
37. Hamilton CM, Strader LC, Pratt JG, Maiese D, Hendershot T, Kwok RK, *et al.* (2011): The PhenX Toolkit: Get the Most From Your Measures. *American Journal of Epidemiology* 174: 253–260.
38. Glynn LM, Stern HS, Howland MA, Risbrough VB, Baker DG, Nievergelt CM, *et al.* (2019): Measuring novel antecedents of mental illness: the Questionnaire of Unpredictability in Childhood. *Neuropsychopharmacology* 44: 876–882.

39. Achenbach TM. (2000): Child Behavior Checklist. *Encyclopedia of Psychology, Vol. 2.* Washington, DC, US: American Psychological Association, pp 69–70.
40. Bernstein DP, Fink L, Handelsman L, Foote J, Lovejoy M, Wenzel K, Sapareto E, Ruggiero J (1994): Initial reliability and validity of a new retrospective measure of child abuse and neglect. *The American Journal of Psychiatry* 151: 1132-1136.
41. Steinberg AM, Brymer MJ, Decker KB, Pynoos RS (2004): The University of California at Los Angeles post-traumatic stress disorder reaction index. *Current Psychiatry Reports* 6: 96–100.
42. Foa EB, Johnson KM, Feeny NC, Treadwell KRH (2001): The Child PTSD Symptom Scale: A Preliminary Examination of its Psychometric Properties. *Journal of Clinical Child & Adolescent Psychology* 30: 376–384.
43. Weiss PDC, Chorpita BF (2015): Revised Children's Anxiety and Depression Scale.
44. Zimet G, Dahlem N, Zimet S, Farley G (1988): The Multidimensional Scale of Perceived Social Support. *Journal of Personality Assessment* 52: 30–41.
45. Berry JO, Jones WH (1995): The Parental Stress Scale: Initial Psychometric Evidence. *Journal of Social and Personal Relationships* 12: 463–472.
46. Elgar FJ, Waschbusch DA, Dadds MR, Sigvaldason N (2007): Development and Validation of a Short Form of the Alabama Parenting Questionnaire. *Journal of Child and Family Studies* 16: 243–259.
47. Du Toit S, Haag K, Skeen S, Sherr L, Orkin M, Rudgard WE, *et al.* (2022): Accelerating progress towards improved mental health and healthy behaviours in adolescents living in adversity: findings from a longitudinal study in South Africa. *Psychology, Health & Medicine* 27: 14–26.
48. Snyder CR, Sympson SC, Ybasco FC, Borders TF, Babyak MA, Higgins RL (1996): Development and validation of the State Hope Scale. *Journal of Personality and Social Psychology* 70: 321–335.
49. Wamboldt MZ, Wamboldt FS, Gavin L, Mctaggart S (2001): A Parent–Child Relationship Scale Derived From the Child and Adolescent Psychiatric Assessment (CAPA). *Journal of the American Academy of Child & Adolescent Psychiatry* 40: 945–953.
50. Marci T, Moscardino U, De Carli P, Altoé G (2019): Measuring insecure attachment in middle childhood: Psychometric evaluation of the short form of the Preoccupied and Avoidant Coping Questionnaire. *Personality and Individual Differences 147*: 91–101.
51. Finnegan RA, Hodges EVE, Perry DG (1996): Preoccupied and Avoidant Coping during Middle Childhood. *Child Development* 67: 1318–1328.
52. Jucovy L (2002): *Measuring the Quality of Mentor-Youth Relationships: A Tool for Mentoring Programs. Technical Assistance Packet*. Retrieved May 2, 2024, from https://eric.ed.gov/?id=ED472173
53. Narayan AJ, Rivera LM, Bernstein RE, Harris WW, Lieberman AF (2018): Positive childhood experiences predict less psychopathology and stress in pregnant women with childhood adversity: A pilot study of the benevolent childhood experiences (BCEs) scale. *Child Abuse & Neglect* 78: 19–30.
54. Dweck CS (2006): *Mindset: The New Psychology of Success*. New York, NY, US: Random House, pp x, 276.
55. Gratz KL, Roemer L (2004): Multidimensional Assessment of Emotion Regulation and Dysregulation: Development, Factor Structure, and Initial Validation of the Difficulties in Emotion Regulation Scale. *Journal of Psychopathology and Behavioral Assessment* 26: 41–54.
56. Goodman R (1997): The Strengths and Difficulties Questionnaire: A Research Note. *Journal of Child Psychology and Psychiatry* 38: 581–586.
57. Swanson JM, Schuck S, Porter MM, Carlson C, Hartman CA, Sergeant JA, *et al.* (2012): Categorical and Dimensional Definitions and Evaluations of Symptoms of ADHD: History of the SNAP and the SWAN Rating Scales. *International Journal of Educational and Psychological Assessment* 10: 51–70.
58. Liddle EB, Batty MJ, Goodman R (2009): The Social Aptitudes Scale: an initial validation. *Social Psychiatry and Psychiatric Epidemiology* 44: 508–513.

59. Connor KM, Davidson JRT (2003): Development of a new resilience scale: The Connor-Davidson Resilience Scale (CD-RISC). *Depression and Anxiety* 18: 76–82.
60. Brodzinsky DM, Elias MJ, Steiger C, Simon J, Gill M, Hitt JC (1992): Coping scale for children and youth: Scale development and validation. *Journal of Applied Developmental Psychology* 13: 195–214.
61. Gratz KL (2001): Measurement of Deliberate Self-Harm: Preliminary Data on the Deliberate Self-Harm Inventory. *Journal of Psychopathology and Behavioral Assessment* 23: 253–263.
62. Snyder CR, Hoza B, Pelham WE, Rapoff M, Ware L, Danovsky M, *et al.* (1997): The Development and Validation of the Children's Hope Scale. *Journal of Pediatric Psychology* 22: 399–421.
63. Wagnild G (2009): A Review of the Resilience Scale. *Journal of Nursing Measurement* 17: 105–113.
64. Gullone E, Taffe J (2012): The Emotion Regulation Questionnaire for Children and Adolescents (ERQ–CA): A psychometric evaluation. *Psychological Assessment* 24: 409–417.
65. Millgram Y, Gruber J, Villanueva CM, Rapoport A, Tamir M (2021): Motivations for Emotions in Bipolar Disorder. *Clinical Psychological Science* 9: 666–685.
66. Bach DR, Schächinger H, Neuhoff JG, Esposito F, Salle FD, Lehmann C, *et al.* (2008): Rising Sound Intensity: An Intrinsic Warning Cue Activating the Amygdala. *Cerebral Cortex* 18: 145–150.
67. Tottenham N, Hare TA, Casey BJ (2011): Behavioral Assessment of Emotion Discrimination, Emotion Regulation, and Cognitive Control in Childhood, Adolescence, and Adulthood. *Frontiers in Psychology* 2: 39.
68. Lieberman MD, Eisenberger N, Crockett M, Tom S, Pfeifer J, Way B (2007): Putting Feelings Into Words Affect Labeling Disrupts Amygdala Activity in Response to Affective Stimuli. *Psychological Science* 18: 421–428.
69. Ochsner KN, Bunge SA, Gross JJ, Gabrieli JDE (2002): Rethinking Feelings: An fMRI Study of the Cognitive Regulation of Emotion. *Journal of Cognitive Neuroscience* 14: 1215–1229.
70. Alexander LM, Escalera J, Ai L, Andreotti C, Febre K, Mangone A, *et al.* (2017): An open resource for transdiagnostic research in pediatric mental health and learning disorders. *Scientific Data* 4: 170181.
71. Lee CS, Cohen SS, Hutchinson S, Tottenham N, Baldassano C (2024, September 15): Neural and verbal responses to attachment-schema narratives differ based on past and current caregiving experiences. bioRxiv, p 2024.09.13.612953.
72. Nikolaidis A, Heleniak C, Fields A, Bloom PA, VanTieghem M, Vannucci A, *et al.* (2022): Heterogeneity in caregiving-related early adversity: Creating stable dimensions and subtypes. *Development and Psychopathology* 34: 621–634.
73. Dam NTV, O'Connor D, Marcelle ET, Ho EJ, Craddock RC, Tobe RH, *et al.* (2017): Data-Driven Phenotypic Categorization for Neurobiological Analyses: Beyond DSM-5 Labels. *Biological Psychiatry* 81: 484–494.
74. Norris FH, Tracy M, Galea S (2009): Looking for resilience: Understanding the longitudinal trajectories of responses to stress. *Social Science & Medicine* 68: 2190–2198.
75. Galatzer-Levy IR, Huang SH, Bonanno GA (2018): Trajectories of resilience and dysfunction following potential trauma: A review and statistical evaluation. *Clinical Psychology Review* 63: 41–55.
76. Klein A, Mensh B, Ghosh S, Tourville J, Hirsch J (2005): Mindboggle: Automated brain labeling with multiple atlases. *BMC Medical Imaging* 5: 7.
77. Garyfallidis E, Brett M, Amirbekian B, Rokem A, Van Der Walt S, Descoteaux M, Nimmo-Smith I (2014): Dipy, a library for the analysis of diffusion MRI data. *Frontiers in Neuroinformatics* 8.
78. Craddock C, Sikka S, Cheung B, Khanuja R, Ghosh S, Yan C, *et al.* (2013): Towards Automated Analysis of Connectomes: The Configurable Pipeline for the Analysis of Connectomes (C-PAC). *Frontiers in Neuroinformatics* 42.
79. Jenkinson M, Beckmann CF, Behrens TEJ, Woolrich MW, Smith SM (2012): FSL. *NeuroImage* 62: 782–790.

80. Vannucci A, Fields A, Heleniak C, Bloom PA, Harmon C, Nikolaidis A, *et al.* (2025): Machine learning for identifying caregiving adversities associated with greatest risk for mental health problems in children. *Nature Mental Health* 1–12.
